# Pan-Cancer Analysis Reveals Disrupted Circadian Clock Associates With T Cell Exhaustion

**DOI:** 10.3389/fimmu.2019.02451

**Published:** 2019-10-24

**Authors:** Yingcheng Wu, Baorui Tao, Tianyang Zhang, Yihui Fan, Renfang Mao

**Affiliations:** ^1^Laboratory of Medical Science, School of Medicine, Nantong University, Jiangsu, China; ^2^Department of Pathophysiology, School of Medicine, Nantong University, Jiangsu, China; ^3^Department of Immunology, School of Medicine, Nantong University, Jiangsu, China

**Keywords:** circadian clock, CLOCK, BMAL1, PD-L1, immune evasion

## Abstract

Although dysfunctional circadian clock has emerged as a hallmark of cancer, fundamental gaps remain in our understanding of the underlying mechanisms involved. Here, we systematically analyze the core genes of the circadian clock (CLOCK, ARNTL, ARNTL2, NPAS2, NR1D1, NR1D2, CRY1, CRY2, RORA, RORB, RORC, PER1, PER2, and PER3) across a broad range of cancers. To our surprise, core negative regulators (PER1, PER2, PER3, CRY1, and CRY2) are consistently downregulated, while core positive regulators show minimal alterations, indicating disrupted circadian clock in cancers. Such downregulation originates from copy number variations where heterozygous deletion predominates. The disrupted circadian clock is significantly associated with patient outcome. Further pathway enrichment analysis suggests that the circadian clock widely impacts 45 pathways such as the Ras signaling pathway and T cell receptor signaling pathway. By using state-of-the-art immune cell deconvolution and pathway quantification, we demonstrate that abnormal circadian clock contributes to T cell exhaustion and global upregulation of immune inhibitory molecules such as PD-L1 and CTLA-4. In summary, the rhythm of the circadian clock is disrupted in cancers. Abnormal circadian clock linked with immune evasion may serve as a potential hallmark of cancer.

## Introduction

Life on earth utilizes internal timing systems such as “circadian clocks” to adapt and respond optimally to daily environmental cycles (1). In mammals, the circadian clock is coordinated by a hierarchical system in which a brain clock located in the hypothalamic suprachiasmatic nucleus (SCN) acts as a master pacemaker to synchronize the peripheral clocks of individual cells (2–4). Local cellular clocks maintain autonomous rhythms and respond to intermittent input from the SCN to form a resonant network (2–4). At the molecular level, the circadian clock in mammals is controlled by three cell-autonomous feedback loops. The first loop includes two activators (CLOCK and BMAL1) and four repressors (PER1, PER2, CRY1, and CRY2) (5). The activation of heterodimer CLOCK:BMAL1 occurs in the daytime, leading to transcription and accumulation of the Per and Cry genes in the late afternoon or evening. PER and CRY proteins translocate into the nucleus at night, where they interact with CLOCK:BMAL1 and block the function of CLOCK:BMAL1 as well as their own transcription (6–8). In another loop, CLOCK/ARNTL is alternately stimulated and repressed by its transcription targets, including RORA/RORB/RORC and REV-ERBs. CLOCK:BMAL1 also drives a loop containing transcription factors such as DBP, TEF, and HLF, which interact with sites containing D-boxes with the repressor NFIL3 driven by the REV-ERB/ROR loop. NFIL3 in turn represses DBP to regulate a rhythm in the RORA/RORB/RORC nuclear receptors (5). Thus, the imbalance change between positive and negative regulators will disrupt the circadian rhythms and is associated with diseases.

Circadian rhythms have impacts on diverse physiological processes, including but not limited to the immune system, the cardiovascular system, and energy metabolism (9). In particular, circadian oscillators participate in the development and specification of innate immunity through the generation of a daily rhythm in the synthesis of cytokines, phagocytosis, and migration (10). However, the role of circadian rhythm inside the cancer microenvironment ecosystem is much less well-understood (11). We hence hypothesize that abnormal circadian clock in cancer cells may affect anti-tumor immunity as well as cancer development in the tumor microenvironment. We seek to address the link between circadian clock dysfunction and the cancer microenvironment.

Escaping circadian regulation is believed to be an emerging hallmark of cancer and tightly associates with patient survival (12, 13). Strong epidemiological evidence links circadian disruption with cancers (14, 15), and enhancing core circadian gene expression will induce the death of cancer cells (16). However, under such a scenario, both enhancing the circadian clock and targeting the circadian clock (16) will restrict cancer development. The seemingly counterintuitive relationship posed by prior studies raises a critical need to systematically understand the link between circadian clock and cancers. Defining the circadian clock-associated pathways in cancer will deepen our knowledge of how cancer cells reprogram themselves.

Here, by using high-throughput sequencing data on thousands of samples, we surprisingly observe that the circadian clock is widely disrupted in cancers. We comprehensively define pathways triggered by disrupted circadian clock and demonstrate that circadian clock tightly associates with immune escape. Our findings provide new insights linking dysfunctional circadian clock with tumor development and anti-tumor immunity.

## Methods and Materials

### Data Sets, Data Availability, and Software Availability

We retrieved Level 3 data including gene expression (raw counts and TPM), survival time, vital status, copy number variation data, and single nucleotide variation from the Cancer Genome Atlas (https://portal.gdc.cancer.gov). The direct website links for downloading each dataset are listed in Table S4. ChIP-seq of H3K4me3 was fetched from the NCBI Sequence Read Archive (https://www.ncbi.nlm.nih.gov/sra) with the accession numbers SRX174721, SRX174722, SRX174723, SRX174724, SRX174725, and SRX174726. All sample information and download links are available in Tables S2–S4. We used RStudio Server for CentOS (Version 1.1.463, https://www.rstudio.com/products/rstudio/download-server/).

### Data Set Inclusion Criteria

For pan-cancer analysis, we used all 11 cancer types with matched normal samples from the Cancer Genome Atlas, including Thyroid Carcinoma (THCA), Kidney Renal Papillary Cell Carcinoma (KIRP), Liver Hepatocellular Carcinoma (LIHC), Stomach Adenocarcinoma (STAD), Breast Invasive Carcinoma (BRCA), Colon Adenocarcinoma (COAD), Uterine Corpus Endometrial Carcinoma (UCEC), Bladder Urothelial Carcinoma (BLCA), Kidney Renal Clear Cell Carcinoma (KIRC), Kidney Chromophobe (KICH), and Prostate Adenocarcinoma (PRAD). We used the 24-h time-series ChIP-seq data of H3K4me3 because H3K4me3 can specifically mark the promoter activity of genes (17) (NCBI Sequence Read Archive with accession numbers SRX174721, SRX174722, SRX174723, SRX174724, SRX174725, and SRX174726).

### ChIP-seq Analysis

We fetched the raw reads from the NCBI Sequence Read Archive by using Aspera (https://asperasoft.com/software/transfer-services/ascp/). We next mapped raw reads to the mouse genome (NCBI mm9) with Applied Biosystems BioScope Version 1.3. Two mapping parameters (ma.to.bam.output.filter = alignment_score, and ma.to.bam.clear.zone = 5) were applied to ensure that the mapped reads in the ma file were limited to “unique” reads for the bam file output. We removed PCR duplicates by using Picard MarkDuplicates (https://software.broadinstitute.org/gatk/documentation/tooldocs/4.0.4.0/picard_sam_markduplicates_MarkDuplicates.php). We used deepTools (https://deeptools.readthedocs.io/en/develop/) to generate a bigwig file and used Gviz (deepTools) to visualize ChIP-seq peaks.

### Gene Expression Analysis

We restricted our analysis to cancers with matched tumor-normal pairs (*n* > 10). Fold change (FC) equals the mean value of tumor gene expression divided by the mean value of normal sample gene expression. Genes with FC > 2 and FDR <0.05 were regarded as significantly differentially expressed genes. We used Deseq2 (https://bioconductor.org/packages/release/bioc/html/DESeq2.html) in R to perform the differential gene expression analysis.

### Survival Analysis

We downloaded patient clinical data (overall survival, overall survival state, progression-free interval, progression-free interval state, disease-free interval, disease-free interval state, disease-specific survival, and disease-specific survival state) and matched them with RNA-seq data for each sample. Samples with no available data were removed from the analysis. Only samples of primary tumors were included for analysis to avoid bias. For survival analysis, we limited patient survival time to 5 years. We separated patients into a high-risk group and a low-risk group according to each gene expression. Only primary tumors were included for analysis. The *P*-value was computed through a log-rank test. We used the survival package (https://cran.r-project.org/web/packages/survival/index.html) and survminer package (https://cran.r-project.org/web/packages/survminer/index.html) in R.

### Copy Number Variation Analysis

We processed the raw copy number variation (CNV) data by GISTIC (18). Only genes with >5% CNV changes in an individual patient were included for analysis in each cancer. We computed the Pearson coefficient of paired mRNA expression and CNV raw data by using the cor.test in R. The *P*-value was adjusted by FDR. We applied a similar pipeline to that previously described (19). The CNV was divided into 2 subtypes, heterozygous CNV and homozygous CNV, which represent the occurrence of CNV on only one chromosome or both.

### Single Nucleotide Variation Analysis

We used maftools (https://bioconductor.org/packages/release/bioc/html/maftools.html) in R to generate the single nucleotide variation (SNV) summary and oncoplot waterfall plot (20). We show genes with an overall mutation proportion of over 10%. The SNV percentage equals the number of mutated samples divided by the total number of samples. The darker the color, the higher the frequency of variation. The number in each cell represents the sample size with each mutated gene in each cancer. The SNVs were divided into missense mutations, nonsense mutations, multiple hits, frameshift insertions, frameshift deletions, splice sites, and in-frame deletions.

### Gene Set Enrichment Analysis

We first retrieved the pan-cancer gene expression profile (TPM, log-transformed). We performed a correlation analysis between each circadian clock gene and all other genes. We next ranked the Spearman's rho estimates and performed gene set enrichment analysis based on their rankings. We used clusterProfiler (21) to analyze the enriched pathways. We used the KEGG signaling pathway gene sets (downloaded from MSigDB, http://software.broadinstitute.org/gsea/msigdb/genesets.jsp). For the 14 core circadian clock genes, we ranked the most overlapped enriched pathways and visualized them when over five pathways were simultaneously enriched. Selected immune-related ranking plots for the 14 core circadian clock genes are shown. To filter the common enriched pathways in the 14 circadian clock genes, we ranked the gene set enrichment analysis enrichment score of each pathway. Enriched pathways in 5 or more genes were regarded as circadian clock-related pathways. We used pheatmap (https://cran.r-project.org/web/packages/pheatmap/index.html) in R to visualize the top-ranked enriched pathways.

### Immune Cell Deconvolution

We used xCell (22) to infer the immune cell and stroma cell infiltration level from the bulk RNA-seq data. We used the pre-calculated immune cell deconvolution of TCGA data from xCell (22).

### Pathway Activity Quantification

We used the PARADIGM (23) inferred patient-specific pathway activities (http://api.gdc.cancer.gov/data/7d4c0344-f018-4ab0-949a-09815f483480). RNA-seq and copy number data were used for input. The T cell anergy score was defined by T_cell_anergy_.abstract, and the circadian clock integrated score was defined by ARNTL.CLOCK.NPAS2.CRY.PER_.complex.

### Statistical Analysis

For differential gene analysis, genes with FC > 2 and FDR < 0.05 were regarded as significantly differentially expressed genes. In survival analysis, we used the log-rank test and considered a *P*-value of <0.05 as indicating statistical significance. For the correlation between CNV and gene expression, we conducted a correlation analysis and show the FDR-adjusted *P*-value and the Pearson R estimate.

### Code Availability

Codes for the data analysis are available on GitHub (https://github.com/fun-science-club-ntu/CLOCK).

## Results

### Disrupted Circadian Clock in Cancers and Its Association With Patient Outcomes

**Figure 1:**
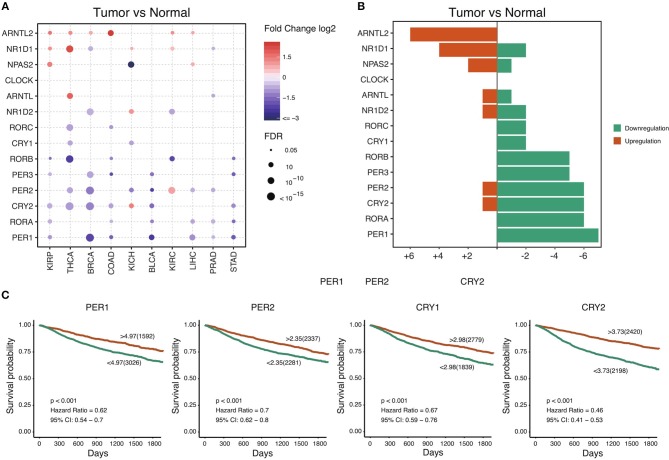
Core circadian clock genes are dysregulated in cancers. **(A)** The mRNA difference between normal samples and tumor samples. Only significant differential expressed genes were shown. **(B)** The summary of mRNA alteration of core circadian clock genes. **(C)** Dysfunction of circadian clock contributes to poor prognosis in cancers.

The circadian clock is cell-autonomous, and its timekeeping arises from a set of transcription–translation feedback loops (TTFLs) (24). In order to achieve a systematic understanding of circadian clock in cancer, we define a core subset of clock family genes including 7 positive regulators (CLOCK, NPAS2, ARNTL, ARNTL2, RORA, RORB, and RORC) and 7 negative regulators (PER1, PER2, PER3, CRY1, CRY2, NR1D1, and NR1D2) (Table S1) (13). We retrieved the transcriptome and genome profile of cancers from the Cancer Genome Atlas (for sample description, see Table S2). Among 716 samples across 29 cancer types, we interestingly observe that most of the negative regulators, such as PER1, PER2, PER3, CRY1, CRY2, and NR1D2, are down-regulated in a wide range of cancers (Figures 1A,B). However, the core positive regulators are upregulated (ARNTL2) or unchanged (CLOCK and ARNTL). This observation suggests that, in cancer, the circadian clock loses its daily rhythmic activity cycle. Next, we determine whether the observed transcriptional changes are associated with patient outcomes. To our surprise, the expression of all individual genes predicts patient outcomes. Patients with high expression of the majority of the negative regulators show a good prognosis compared with patients with low expression (Figure 1C and Figure S1). High expression of the positive regulators including ARNTL2 and NPAS2 predicts poor outcomes (Figure 1C and Figure S1). Collectively, our findings demonstrate that cancer cells widely downregulate negative regulators and possibly shift the rhythm of the circadian clock. The dysregulation of the circadian clock is highly associated with patient outcomes.

### Heterozygous Deletion Results in Dysregulation of the Circadian Clock in Cancers

**Figure 2:**
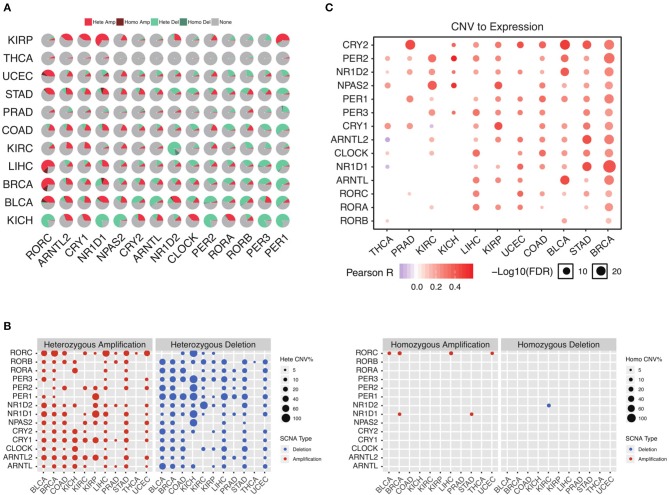
Heterozygous deletion underlies the circadian clock dysregulation. **(A)** The proportion of each copy number variation in cancers. **(B)** Heterozygous and homozygous amplification/deletion of circadian clock genes in cancers. **(C)** Copy number variation strongly associates with gene expression of circadian clock.

To further understand why the core circadian clock genes are transcriptionally dysregulated, we examine the copy number variation in pan-cancer. Consistent with the down-regulation of negative regulators, we observe significant heterozygous deletion of most negative regulators, including PER1, PER2, PER3, CRY2, and NR1D2 (Figure 2A). The upregulated positive regulators, such as ARNTL2 and NPAS2, are frequently heterozygous amplified (Figure 2A). In contrast, the frequencies of homozygous deletion and amplification are extremely low (Figure 2B). Our results indicate that the transcriptional dysregulation of the circadian clock is very likely due to heterozygous deletion or amplification. To further support our conclusion, we conduct a correlation analysis between copy number variation (CNV) and mRNA level. As is shown in Figure 2C, in the majority of cancer tissues, the CNV is associated with the mRNA level. Therefore, the transcriptional dysregulation of the circadian clock in cancer is mainly due to heterozygous deletion or amplification.

### Sequence Alteration Also Contributes to Abnormal Circadian Clock in Cancers

**Figure 3:**
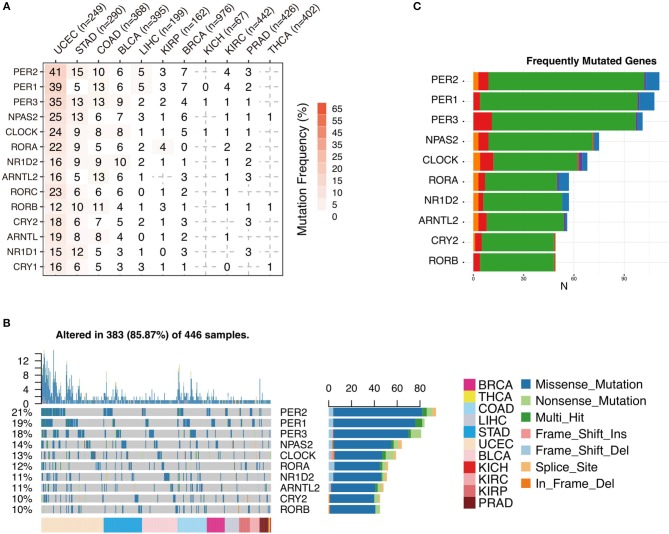
Sequence alteration contributes to abnormal circadian clock. **(A)** Mutation frequency of core circadian clock genes. **(B)** Mutation type and summary of core circadian clock genes in cancers. **(C)** Frequently mutated gene ranking.

To further analyze the status of circadian clock in cancer, besides transcripts, we analyze DNA sequence alteration, which results in abnormal protein function but might not affect mRNA level. We first examine the genomic profile and, surprisingly, find that most of the analyzed genes are frequently mutated (Figure 3A and Figure S2). The mutation rate in several cancers, such as UCEC, STAD, COAD, and BLCA, is very high. In 446 analyzed patients, 383 (85.87%) show at least one mutation (Figure 3B). Missense mutation is a major variant event. The genes PER2, PER1, and PER3 are the most frequently mutated genes (Figure 3C and Figure S2). Our results indicate that besides transcriptional alteration, DNA sequence alteration might also be an important factor that contributes to abnormal circadian clock in cancer.

### Oncogenic Pathways Associated With Circadian Clock in Cancers

**Figure 4:**
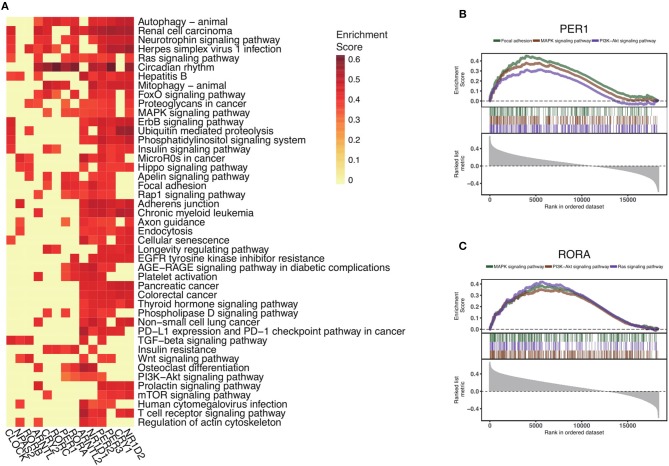
Circadian clock widely impacts signaling pathways in pan-cancer. **(A)** Rankings of pathways linked with the circadian clock in cancers. Signaling pathways were ranked by the frequency of correlation with core clock genes. **(B)** Significantly enriched pathways of PER1 and **(C)** RORA. Gene set enrichment analysis of Spearman's rho rankings were performed.

To explore the functional impact of altered core clock genes in cancers, we perform gene set enrichment analysis of the 14 core circadian rhythm genes. First, we compute the Spearman's rho estimate between those core circadian genes and the transcriptome in the pan-cancer setting. We next conduct gene set enrichment analysis of the 14 circadian clock genes and rank all significant pathways (Methods) (Figure 4A). Interestingly, the most down-regulated genes (RORA and PER1) are associated with the MAPK signaling pathway, PI3K-AKT signaling pathway, and Ras signaling pathway (Figures 4B,C). In total, we identify 45 of the most circadian clock-enriched pathways (Figure 4A, Figure S3). Among all of the oncogenic pathways, the Ras signaling pathway and autophagy pathways are the most affected pathways. In summary, we define the common signaling pathways associated with circadian clock in cancers. Abnormal circadian clock in cancer might play a fundamental role in cancer development.

### Abnormal Circadian Clock Contributes to T Cell Anergy and Upregulation of Immune Checkpoint Molecules

**Figure 5:**
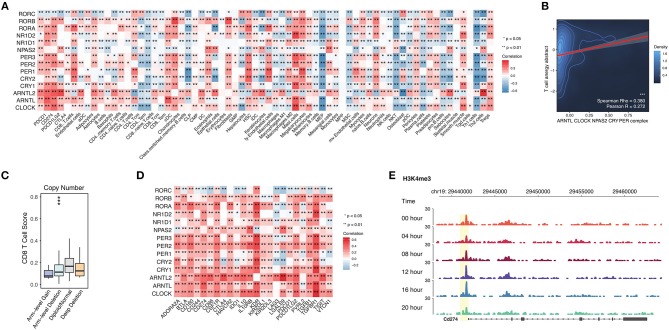
Abnormal circadian clock contributes to T cell anergy and upregulation of immune checkpoint molecules. **(A)** Core circadian clock genes widely link with infiltrated immune cells and stroma cells in cancers. **(B)** Circadian clock associates with T cell anergy. T cell anergy and the circadian clock complex were quantified by PARADIGM (Methods). **(C)** Deletion of PER1 contributes to the reduced CD8 T cell infiltration level in stomach adenocarcinoma. **(D)** Circadian clock genes are positively correlated with immune inhibitory molecules. **(E)** The promoter activity of PD-L1 (CD274) is controlled by the circadian rhythm.

When analyzing the circadian clock-enriched pathways, we interestingly find that many immune-related pathways are enriched. The identified immune-related pathways include the MAPK signaling pathway, NFkB signaling pathway, TGF-beta signaling pathway, PD-L1 expression and PD-1 checkpoint pathway in cancer, T cell receptor signaling pathway, TNF signaling pathway, RIG-I like receptor signaling pathway, and JAK-STAT signaling pathway. This suggests that the circadian clock might have wide effects on tumor immunity. We hence apply the deconvolution-based immune signature (Methods) to quantify this potential association in RNA-seq pan-cancer data precisely. We evaluate the correlation between core clock gene expression and CD8 T cells, CD4 T cells, B cells, dendritic cells, macrophages, neutrophils, etc. (Figure 5A). Circadian clock is positively associated with Treg cells and Mast cells, but it negatively correlated with Th2 cells, Th1 cells, NKT cells, CD8 Tem cells, CD8 naïve T cells, and CD4 Tem cells (Figure 5A). These data reveal that the circadian clock widely affects tumor immunity and is generally associated with an immune evasion phenotype. This speculation is supported by another approach that shows that the integrated circadian clock pathway correlates with T cell anergy (Figure 5B). In order to further quantify the possible causal relationship, we stratify patients into groups of circadian gene deletion/normal/amplification patients (Methods). For example, in stomach adenocarcinoma, deep deletion and arm-level deletion of CLOCK give rise to a downregulated level of CD8 T cells (Figure 5C).

Persistently elevated expression of inhibitory checkpoints such as CD274 (PD-L1), PD-L2, PD-1, CTLA-4, and TGFB1 (TGF-beta1) can result in T cell anergy. Thus, we tested the correlation between circadian clock genes and known immune inhibitors defined by a previous study (25). Interestingly, known inhibitory checkpoints such as CD274 (PD-L1), PD-L2, PD-1, CTLA-4, and TGFB1 (TGF-beta1) are positively correlated with all core clock genes (Figure 5D). To further understand whether PD-L1 is rhythmic, we use ChIP-seq data for the H3K4me3 antibody (Methods), because H3K4me3 histone acetylation marks the promoter activity (17). The ChIP-seq of H3K4me3 antibody in mouse liver cells shows that the promoter activity of PD-L1 is rhythmic (Figure 5E). Together, our results demonstrate that the circadian clock has a direct regulatory effect on PD-L1.

## Discussion

The field of chrono-oncology has encountered a paradox: enhancing the circadian clock will induce the death of cancer cells (26, 27) while targeting it will also restrict cancer development (16). This is a sober reminder that the circadian clock program intersects with cancer biology. However, the identity of such programs, how they are generated, and how they trigger oncogenesis are poorly defined. Here, by analyzing 14 core genes of the circadian clock in thousands of patients and applying state-of-the-art algorithms, we achieved a more comprehensive understanding regarding change in circadian clock in cancer. Across different cancers, we observed that the negative regulatory genes are downregulated while the positive regulatory genes, such as BMAL1 and CLOCK, show minimal alterations. This unexpected finding suggests that, unlike normal cells, cancer cells reprogram their circadian rhythms. We next define the oncogenic pathways linked with such alteration. Dysregulated circadian clock profoundly affects the cancer microenvironment and triggers the immune evasion phenotype. Our data support the suggestion that restoring circadian rhythms to normal or completely losing the function of the circadian clock might be a promising strategy for controlling cancer development.

To date, the importance of individual circadian clock component alteration has been documented in several cancer types (16, 28–33); however, a system-level investigation has not been attempted. Our findings demonstrate that, partly consistent with a recent report (34), almost all of the key components of the circadian clock show transcriptional or genetic alteration, indicating that abnormal circadian clock is a common mechanism of oncogenesis. Besides expression, all of the analyzed components show a high proportion of mutations. For example, 53 of 101 (53%) samples of microsatellite instability (MSI) colorectal cancers (CRCs) have been shown to have mutations involved in CLOCK exon 8 (35). In agreement with this, we demonstrate that CLOCK is frequently mutated across cancers despite its minimal change in the mRNA level. We identified a panel of mutations on the 14 core components, but the functional consequences of such mutations are still obscure. By considering the mutation frequency and changed expression, abnormal circadian clock should be more common than previously thought. Detailed functional exploration of each mutation will provide useful information to achieve a better understanding of the function of circadian clock in cancer.

Although our results highlight the frequent abnormal circadian clock in cancers, its underlying mechanism in carcinogenesis is largely unknown. A recent computational paper demonstrated that the circadian clock is linked with hypoxia signaling across a broad spectrum of cancer types (34). Here, we found that 9 of 14 core circadian clock genes associated with the Ras signaling pathway. In accordance with the previous report, another independent group highlighted that the Ras pathway mediated the deregulation of circadian clock in cancer (36). These data indicate that the Ras pathway might be an important pathway upstream of the circadian clock for preventing cancer development. We also identify new potential circadian clock-related pathways such as the ErbB signaling pathway. Future *in vivo* efforts are needed to further confirm the complex functions of circadian clock in cancers.

Outside the tumor, the immune system is believed to under circadian control (37). Inside the tumor, however, for the tumor microenvironment-resident immune cells, very little is known about the role of the circadian clock. One interesting and significant finding is the close relationship between circadian clock and anti-tumor immunity. A broad range of our identified circadian clock-associated pathways are tightly related to immunity, especially to T cell-mediated immune responses such as PD–L1 expression and the PD−1 checkpoint pathway in cancer. We are particularly interested in the PD-L1/PD-1 checkpoint pathway because it is clinically relevant. Further analysis reveals that CLOCK and BAML1 are strongly associated with checkpoint inhibitor molecules such as PD-L1 and CTLA-4. The circadian clock-induced high expression of immune inhibitory checkpoints might contribute to T cell anergy, which is a phenomenon that occurs due to persistent T cell activation. However, at the molecular level, it is still largely unknown how the circadian clock induces the expression of PD-L1. The circadian clock might play an important role in immune evasion and affect the response to immune checkpoint blockade. Investigations regarding the circadian clock and immune checkpoints will provide new insights to boost immunotherapy.

In a nutshell, we have demonstrated the dysregulation of core circadian clock genes and define the regulatory origin of such downregulation. We further screened the circadian clock-associated pathways and observed that the circadian clock widely associates with tumor-infiltrated immune cells, especially T cell exhaustion. Our results collectively provide new insights into the regulation of inhibitory checkpoints and immune cell infiltration. Chronotherapy strategy might be considered to boost the use of immune checkpoint blockade.

## Data Availability Statement

The raw data supporting the conclusions of this manuscript will be made available by the authors, without undue reservation, to any qualified researcher.

## Author Contributions

YF and RM: conceptualization. YW, BT, and TZ: data curation. YW: formal analysis. YF and RM: funding acquisition. YW, YF, and RM: investigation and methodology. YF and RM: project administration, resources, supervision, and validation. YW: visualization. YW, YF, and RM: writing—original draft and writing—review and editing.

### Conflict of Interest

The authors declare that the research was conducted in the absence of any commercial or financial relationships that could be construed as a potential conflict of interest.

## Supplementary Material

Figure S1 Circadian clock genes stratified by patient vital status including **(A)** overall survival (OS), **(B)** progression-free interval (PFI), **(C)** disease-free interval (DFI), and **(D)** disease-specific survival (DSS).

Figure S2 Single nucleotide polymorphism of circadian clock genes in cancers. **(A)** Variant types of clock genes. **(B)** Variant classifications of clock genes. **(C)** Single nucleotide polymorphism class of clock genes. **(D)** Variants per sample of clock genes. **(E)** Summary of variant classification.

Figure S3 Gene set enrichment analysis of signaling pathways among all core circadian clock genes. Selected enriched pathways of **(A)** RORC, **(B)** NR1D2, **(C)** CRY2, **(D)** RORB, **(E)** NR1D1, **(F)** CLOCK, **(G)** PER3, **(H)** CRY2, **(I)** ARNTL, **(J)** PER2, **(K)** NPAS2, and **(L)** ARNTL in pan-cancer.

Table S1 List of core circadian clock genes.

Table S2 Patient characteristics used for analysis.

Table S3 Abbreviations of cancer types and datasets used for analysis.

Table S4 Open resource download link used for analysis.
