# Supplementary figures and images for: Pan-Cancer Analysis Reveals Disrupted Circadian Clock Associates With T Cell Exhaustion
Source: Front Immunol. 2019 Oct 24;10:2451. doi: 10.3389/fimmu.2019.02451 (PMC6821711; doi:10.3389/fimmu.2019.02451)

A OS

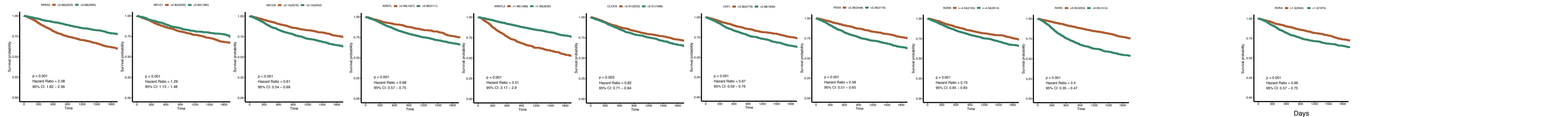

B PFI

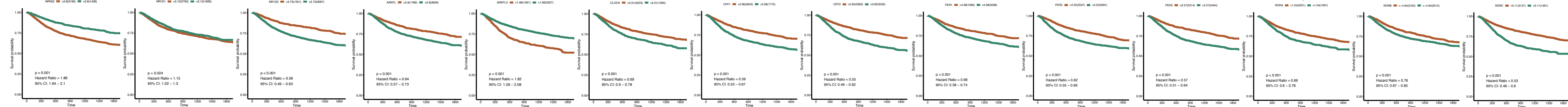

C DFI

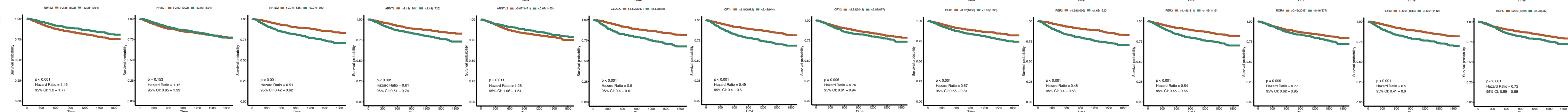

D DSS

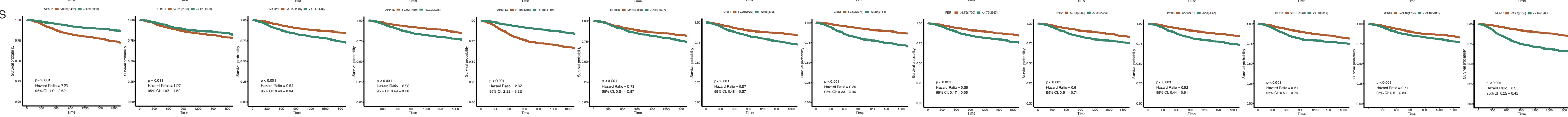

Supplement: Figure S1 — Circadian clock genes stratified by patient vital status including (A) overall survival (OS), (B) progression-free interval (PFI), (C) disease-free interval (DFI), and (D) disease-specific survival (DSS). [file Image_1.PDF]

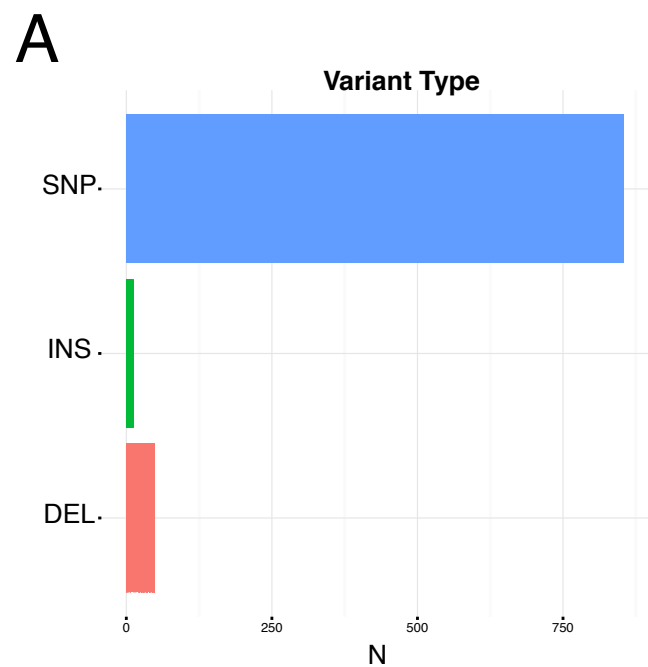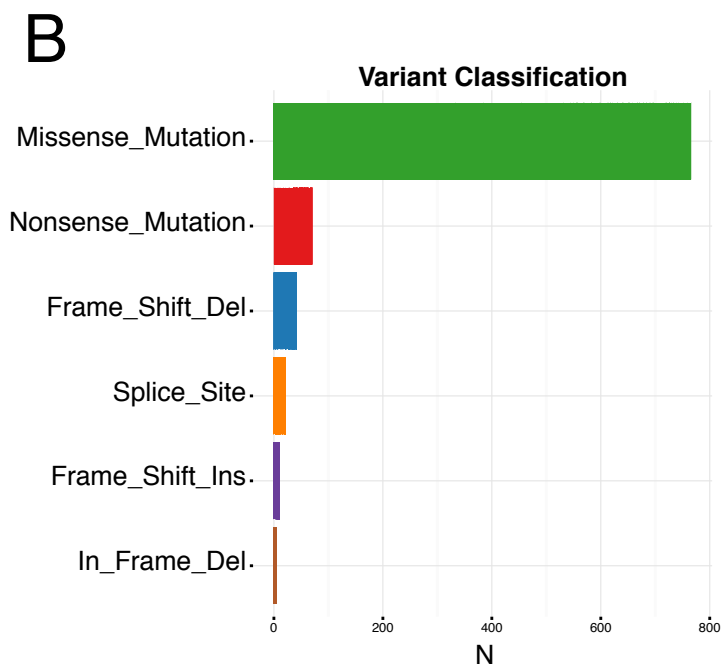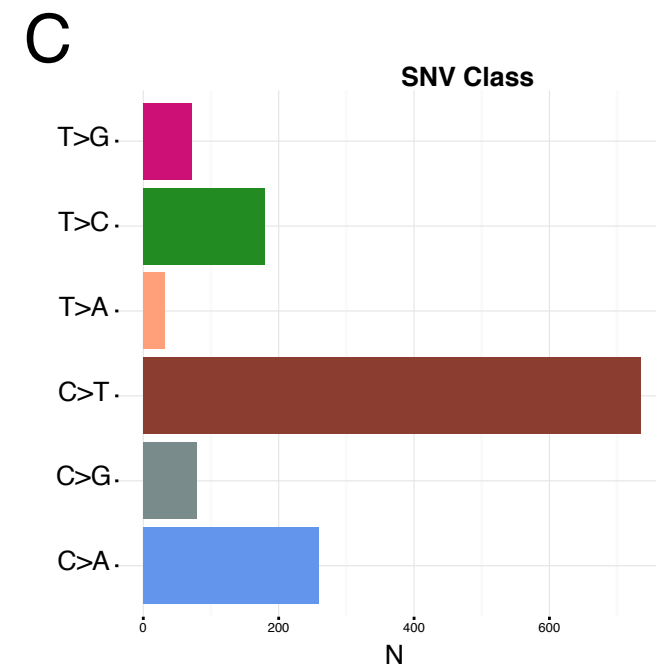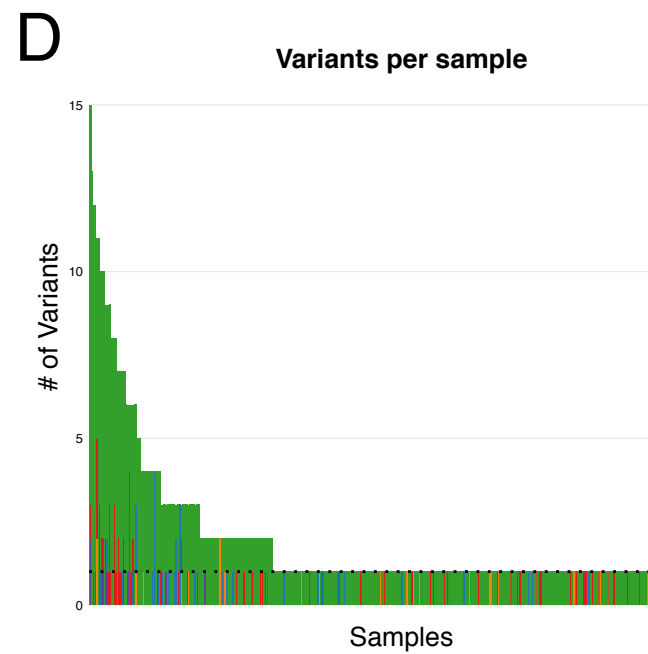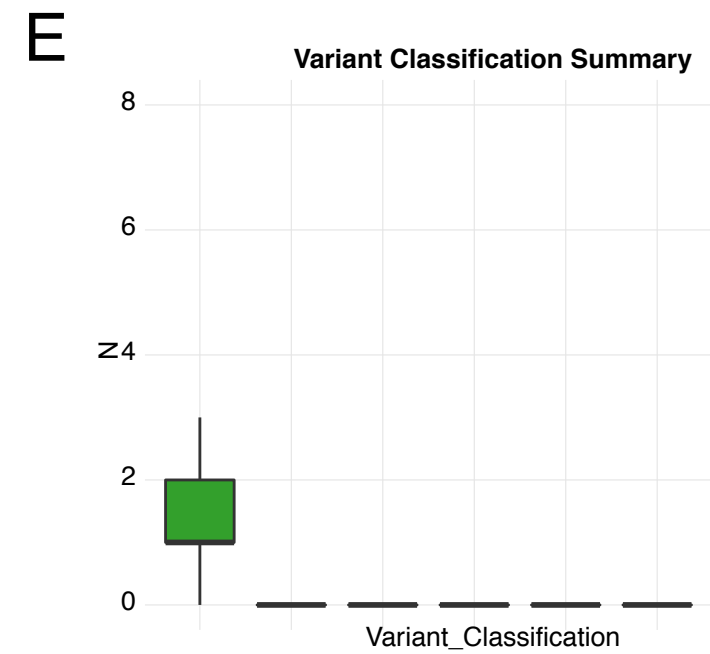

Supplement: Figure S2 — Single nucleotide polymorphism of circadian clock genes in cancers. (A) Variant types of clock genes. (B) Variant classifications of clock genes. (C) Single nucleotide polymorphism class of clock genes. (D) Variants per sample of clock genes. (E) Summary of variant classification. [file Image_2.PDF]

A

RORC

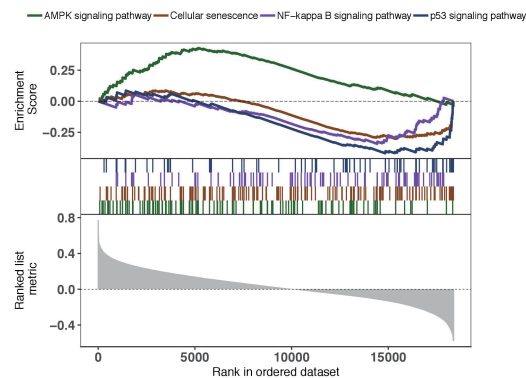

B

NR1D2

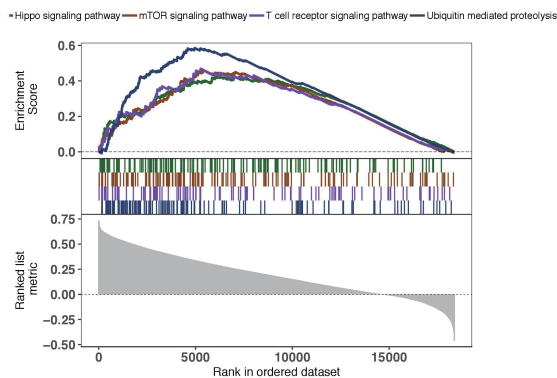

C

CRY2

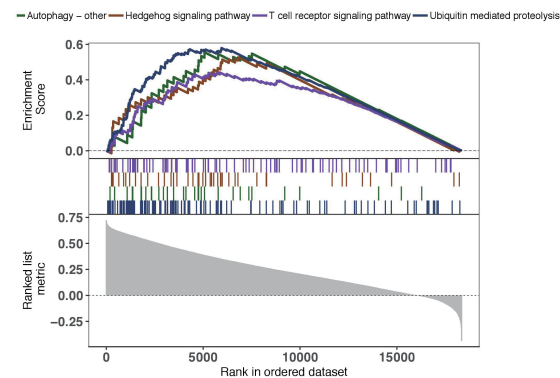

D

RORB

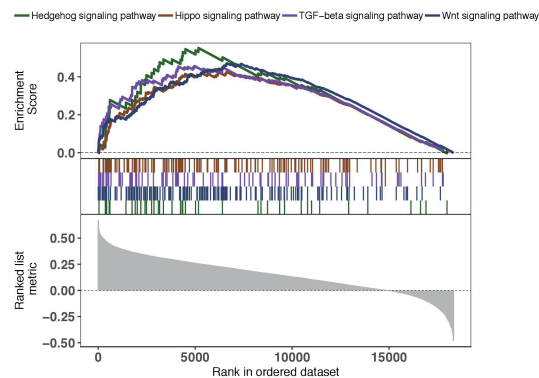

E

NR1D1

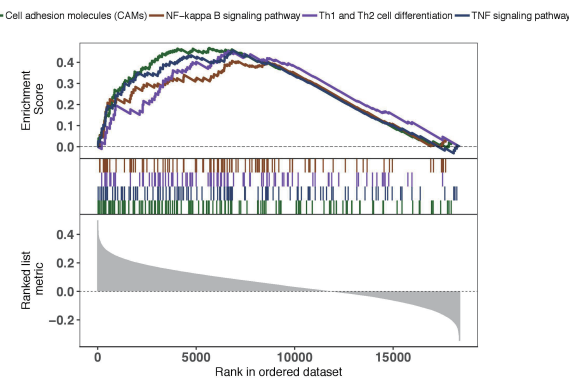

F

CLOCK

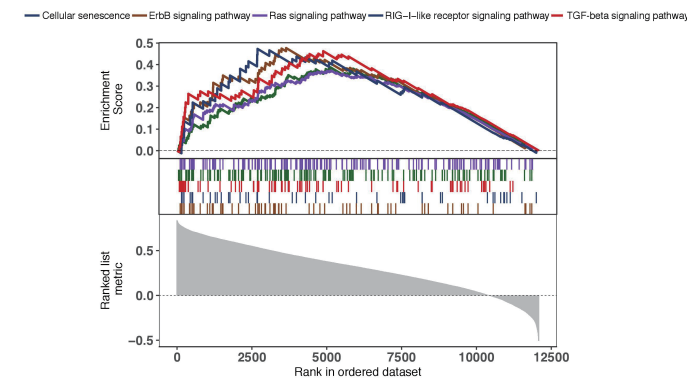

G

PER3

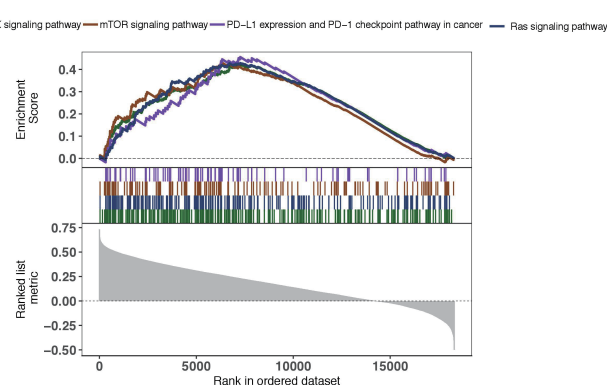

H

CRY2

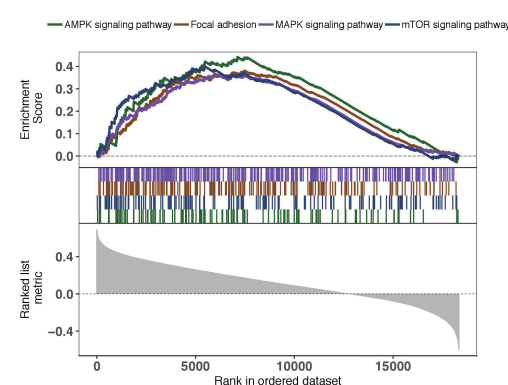

I

ARNTL

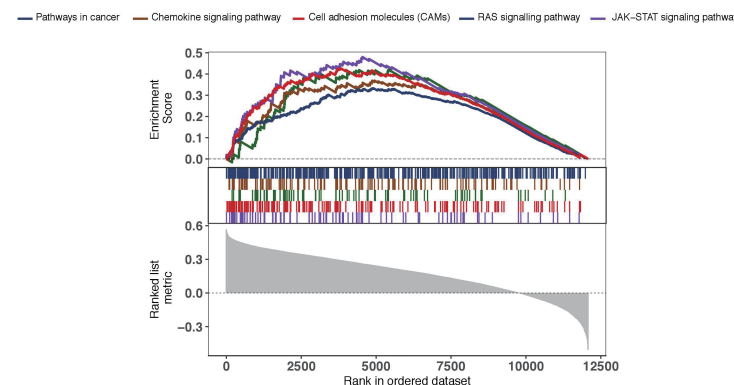

J

PER2

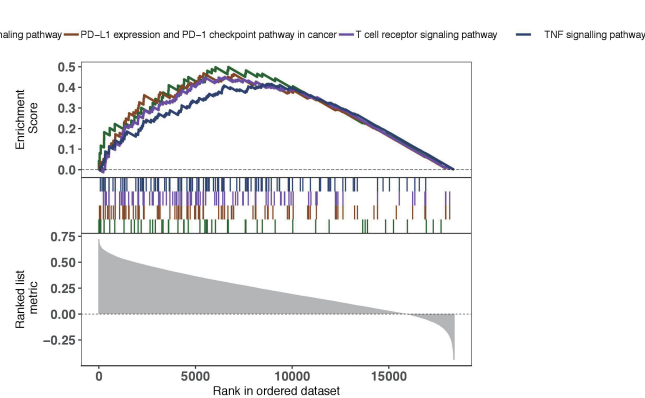

K

NPAS2

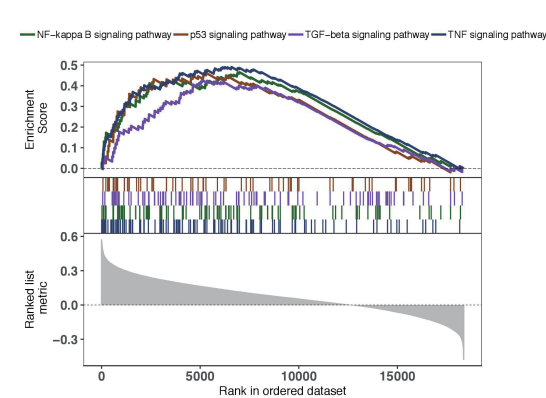

L

ARNTL2

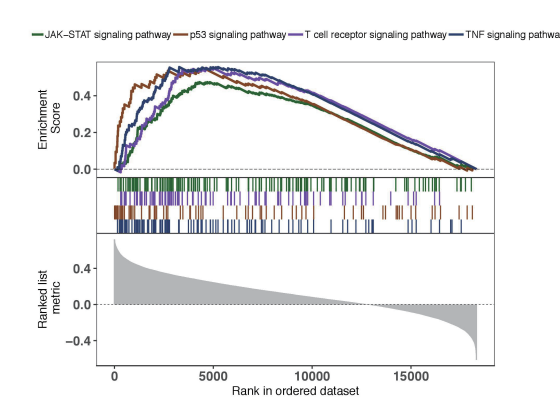

Supplement: Figure S3 — Gene set enrichment analysis of signaling pathways among all core circadian clock genes. Selected enriched pathways of (A) RORC, (B) NR1D2, (C) CRY2, (D) RORB, (E) NR1D1, (F) CLOCK, (G) PER3, (H) CRY2, (I) ARNTL, (J) PER2, (K) NPAS2, and (L) ARNTL in pan-cancer. [file Image_3.PDF]
