# Supplementary material for: Pan-Cancer Analysis Reveals Disrupted Circadian Clock Associates With T Cell Exhaustion
Source: Front Immunol. 2019 Oct 24;10:2451. doi: 10.3389/fimmu.2019.02451 (PMC6821711; doi:10.3389/fimmu.2019.02451)
Supplement: Table S1 — List of core circadian clock genes. [file Table_1.docx]

| Symbol | Ensembl | Name | Group |
| --- | --- | --- | --- |
| CLOCK | ENSG00000134852 | clock circadian regulator | Positive |
| ARNTL | ENSG00000133794 | aryl hydrocarbon receptor nuclear translocator-like | Positive |
| ARNTL2 | ENSG00000029153 | aryl hydrocarbon receptor nuclear translocator-like 2 | Positive |
| NPAS2 | ENSG00000170485 | neuronal PAS domain protein 2 | Positive |
| NR1D1 | ENSG00000126368 | nuclear receptor subfamily 1, group D, member 1 | Negative |
| NR1D2 | ENSG00000174738 | nuclear receptor subfamily 1, group D, member 2 | Negative |
| CRY1 | ENSG00000008405 | cryptochrome circadian clock 1 | Negative |
| CRY2 | ENSG00000121671 | cryptochrome circadian clock 2 | Negative |
| PER1 | ENSG00000179094 | period circadian clock 1 | Negative |
| PER2 | ENSG00000132326 | period circadian clock 2 | Negative |
| PER3 | ENSG00000049246 | period circadian clock 3 | Negative |
| RORA | ENSG00000069667 | RAR-related orphan receptor A | Positive |
| RORB | ENSG00000198963 | RAR-related orphan receptor B | Positive |
| RORC | ENSG00000143365 | RAR-related orphan receptor C | Positive |

**Table S1. List of core circadian clock genes.**
