# Supplementary material for: Pan-Cancer Analysis Reveals Disrupted Circadian Clock Associates With T Cell Exhaustion
Source: Front Immunol. 2019 Oct 24;10:2451. doi: 10.3389/fimmu.2019.02451 (PMC6821711; doi:10.3389/fimmu.2019.02451)
Supplement: Table S2 — Patient characteristics used for analysis. [file Table_2.DOCX]

|  | Alive (n=3693) | Dead with tumor (n=571) | Dead tumor free (n=260) | Total (n=4524) |
| --- | --- | --- | --- | --- |
| female | 1989 (53.9\%) | 247 (43.3\%) | 131 (50.4\%) | 2367 (52.3\%) |
| male | 1704 (46.1\%) | 324 (56.7\%) | 129 (49.6\%) | 2157 (47.7\%) |
| Mean (SD) | 58.7 (13.4) | 63.1 (12.4) | 69.3 (11.3) | 59.9 (13.4) |
| Median [MIN, MAX] | 60 [15,90] | 63 [18,90] | 72 [31,90] | 61 [15,90] |
| Other/unclear | 712 (19.3\%) | 53 (9.3\%) | 17 (6.5\%) | 623 (13.8\%) |
| Stage I | 1072 (29.0\%) | 39 (6.8\%) | 77 (29.6\%) | 1084 (24.0\%) |
| Stage II | 1033 (28.0\%) | 99 (17.3\%) | 74 (28.5\%) | 1114 (24.6\%) |
| Stage III | 708 (19.2\%) | 187 (32.7\%) | 83 (31.9\%) | 906 (20.0\%) |
| Stage IV | 168 (4.5\%) | 193 (33.8\%) | 9 (3.5\%) | 349 (7.7\%) |

**Table S2. Patient characteristics used for analysis**
