# Supplementary material for: Pan-Cancer Analysis Reveals Disrupted Circadian Clock Associates With T Cell Exhaustion
Source: Front Immunol. 2019 Oct 24;10:2451. doi: 10.3389/fimmu.2019.02451 (PMC6821711; doi:10.3389/fimmu.2019.02451)
Supplement: Table S3 — Abbreviations of cancer types and datasets used for analysis. [file Table_3.DOCX]

| Name | Abbreviation | Accession ID |
| --- | --- | --- |
| Thyroid Carcinoma | THCA | TCGA-THCA |
| Kidney Renal Papillary Cell Carcinoma | KIRP | TCGA-KIRP |
| Liver Hepatocellular Carcinoma | LIHC | TCGA-LIHC |
| Stomach Adenocarcinoma | STAD | TCGA-STAD |
| Breast Invasive Carcinoma | BRCA | TCGA-BRCA |
| Colon Adenocarcinoma | COAD | TCGA-COAD |
| Uterine Corpus Endometrial Carcinoma | UCEC | TCGA-UCEC |
| Bladder Urothelial Carcinoma | BLCA | TCGA-BLCA |
| Kidney Renal Clear Cell Carcinoma | KIRC | TCGA-KIRC |
| Kidney Chromophobe | KICH | TCGA-KICH |
| Prostate Adenocarcinoma | PRAD | TCGA-PRAD |

**Table S3. Abbreviations of cancer types and datasets used for analysis.**
