# Supplementary material for: Pan-Cancer Analysis Reveals Disrupted Circadian Clock Associates With T Cell Exhaustion
Source: Front Immunol. 2019 Oct 24;10:2451. doi: 10.3389/fimmu.2019.02451 (PMC6821711; doi:10.3389/fimmu.2019.02451)
Supplement: Table S4 — Open resource download link used for analysis. [file Table_4.DOCX]

| Data | Link |
| --- | --- |
| TCGA RNA-seq | <http://api.gdc.cancer.gov/data/3586c0da-64d0-4b74-a449-5ff4d9136611> |
| TCGA Copy Number | <http://api.gdc.cancer.gov/data/00a32f7a-c85f-4f86-850d-be53973cbc4d> |
| TCGA Mutation | <http://api.gdc.cancer.gov/data/1c8cfe5f-e52d-41ba-94da-f15ea1337efc> |
| TCGA Survival Data | <http://api.gdc.cancer.gov/data/0fc78496-818b-4896-bd83-52db1f533c5c> |
| TCGA Clinical Phenotype | <https://gdc.xenahubs.net/download/GDC-PANCAN/Xena_Matrices/GDC-PANCAN.GDC_phenotype.tsv.gz> |
| TCGA Pathway Activity | <http://api.gdc.cancer.gov/data/7d4c0344-f018-4ab0-949a-09815f483480> |
| H3K4me3 ChIP-seq fastq file (00 hour) | <https://www.ncbi.nlm.nih.gov/sra/SRX174721> |
| H3K4me3 ChIP-seq fastq file (04 hour) | <https://www.ncbi.nlm.nih.gov/sra/SRX174722> |
| H3K4me3 ChIP-seq fastq file (08 hour) | <https://www.ncbi.nlm.nih.gov/sra/SRX174723> |
| H3K4me3 ChIP-seq fastq file (12 hour) | <https://www.ncbi.nlm.nih.gov/sra/SRX174724> |
| H3K4me3 ChIP-seq fastq file (16 hour) | <https://www.ncbi.nlm.nih.gov/sra/SRX174725> |
| H3K4me3 ChIP-seq fastq file (20 hour) | <https://www.ncbi.nlm.nih.gov/sra/SRX174726> |
| xCell | <http://xcell.ucsf.edu/> |
| KEGG gene sets | <http://software.broadinstitute.org/gsea/msigdb/genesets.jsp> |

**Table S4. Open resource download link used for analysis.**
